# Oxidative Damage and Antioxidants as Markers for the Selection of Emersion Hardening Treatments in Greenshell^TM^ Mussel Juveniles (*Perna canaliculus*)

**DOI:** 10.3390/antiox13020198

**Published:** 2024-02-04

**Authors:** Natalí J. Delorme, David J. Burritt, Leonardo N. Zamora, Mena R. V. Welford, Paul M. South

**Affiliations:** 1 Cawthron Institute, Private Bag 2, Nelson 7042, New Zealand; leo.zamora@cawthron.org.nz (L.N.Z.); mena.welford@auckland.ac.nz (M.R.V.W.); paul.south@cawthron.org.nz (P.M.S.); 2 Department of Botany, University of Otago, P.O. Box 56, Dunedin 9054, New Zealand

**Keywords:** green-lipped mussel, oxidative stress, mussel spat, live transport, aquaculture

## Abstract

Transport out of the water is one of the most challenging events for juvenile *Perna canaliculus* and can be a highly inefficient process, with many juveniles subsequently being lost following extended periods of emersion. Hardening techniques offer a possible method for reducing transport-related stress. In this study, different hardening treatments (short, long and intermittent sub-lethal emersion) were used to prepare ~1.2 mm *P.canaliculus* for transport (20 h) and subsequent reoxygenation stress during re-immersion (i.e., recovery). The oxidative stress responses, resettlement behaviour, respiration rates and survival of the mussels after transport and during recovery were all assessed. Short emersion (1 h) as a hardening treatment prior to transport did not cause major stress to the mussels, which maintained respiration at control levels, showed significantly stimulated antioxidant defences during recovery, showed greater resettlement behaviour and remained viable after 24 h of recovery. In comparison, the long and intermittent emersion treatments negatively impacted oxidative stress responses and affected the viability of the mussels after 24 h of recovery. This study showed that exposing juvenile *P.canaliculus* to a mild stress prior to transport may stimulate protective mechanisms, therefore eliciting a hardening response, but care must be taken to avoid overstressing the mussels. Improving the management of stress during the transport of juvenile mussels may be key to minimising mussel losses and increasing harvest production, and biomarkers associated with oxidative stress/antioxidant metabolism could be valuable tools to ensure emersion hardening does not overstress the mussels and reduce survival.

## 1. Introduction

The physiological impacts of emersion on the recovery dynamics of marine organisms have important ramifications for mussel aquaculture industries that routinely expose their stock to different periods of emersion, either to transport stock from sourcing sites or among marine farms, to carry out routine maintenance such as the treatment of biofouling or for crop assessments [1,2,3]. For many sectors, one of the most significant periods of emersion occurs around seeding, when juvenile mussels are transported from their site of origin (i.e., wild spat-catch sites or land-based hatcheries or nurseries) to a marine farm in the ocean [4,5]. Transport operations can be highly inefficient, with many juveniles subsequently being lost due to altered behavioural and physiological characteristics or reduced survival following extended periods of emersion [1,6,7,8].

Previous research has shown that the emersion (48 h) of juvenile *Mytilus edulis* can reduce their byssus production and physiological condition (tissue pH and glucose, glycogen and propionate concentrations) [1]. It is possible that such reductions are linked to stress responses in the juveniles as they respond to and recover from stressors associated with their transport. During transport, environmental conditions such as relative humidity (RH) can vary considerably due to operational processes including packaging or transportation in different vehicles [1,9,10,11]. In wild juvenile mussels, resilience to desiccation stress due to reduced RH increases with size [12,13]. For example, the survival rate of 1–2 mm long *Mytilus trossulus* after a 24 h period of emersion was lower than that of their larger 2–3 mm counterparts [12]. After air exposure or transport, reoxygenation stress can result in a rapid increase in metabolic activity and significant increases in reactive oxygen species (ROS) formation, oxidative damage and the activities of enzymatic antioxidants [14,15,16,17]. In cultured juvenile *Perna canaliculus* (measuring ~1 mm in shell length), resettlement behaviour after transport was reduced when the RH was lower than ~90% during emersion [18]. It has also been described for *P. canaliculus* that when juveniles are emersed, the recovery dynamics, including their oxidative stress response and enzymatic antioxidant activity after re-immersion, vary in relation to the conditions experienced during emersion, often impacting their survival [19].

The endemic Greenshell^TM^ mussel *Perna canaliculus* is the most important aquaculture species in New Zealand, where it also has significant ecological and cultural roles [5,20]. Most (~80%) of the juveniles that are seeded into the New Zealand mussel industry are sourced from the wild, primarily obtained from one region in the far north of the country and typically highly variable in terms of the timing of their delivery, size and condition [5]. The use of these wild-sourced juvenile mussels is inefficient, and most (70–99%) are lost soon after the seeding process, causing a significant constraint to growth in mussel production [5,8,21]. Across the New Zealand mussel-growing industry, less than 1% of the mussels seeded onto marine farms are retained and harvested [22].

Juvenile mussels can be sensitive to transport, handling and heat stresses, potentially affecting their resilience to other stressors and survival after they are seeded onto marine farms [18,23,24,25]. Conditioning or hardening (i.e., brief exposure to sub-lethal stress that results in a transitory increase in tolerance to subsequent stress; definition modified from a review paper [26]) techniques offer possible methods of reducing the effects of transport-related stress. Typically, such techniques involve exposing organisms to elevated stress prior to transportation, stimulating physiological mechanisms that protect against or alleviate cellular damage [27]. In adult *P. canaliculus*, thermal tolerance and survival (i.e., heat hardening) were increased in mussels that were heat-treated prior to thermal challenges [28]. In this example, the heat-treated mussels had significant increases in specific metabolic precursors of antioxidant molecules such as the amino acids glycine, cysteine, glutamic acid and methionine, suggesting a greater capacity to increase their metabolic response to oxidative stress [28]. In *M. edulis*, intertidal acclimatization increased the air exposure tolerance of mussels, shifting the patterns and rates of metabolite accumulation such as the glycolytic enzyme pyruvate kinase [29]. In *M. galloprovincialis*, air exposure was also shown to stimulate the heat shock response by inducing the expression of heat shock proteins [30].

In this study, the effects of pre-transport emersion as a potential hardening method to prepare juvenile *P. canaliculus* for transport from a hatchery or nursery to a sea-based marine farm were investigated. Different hardening treatment times to improve the response of juvenile mussels to the impacts of reoxygenation stress during immersion in seawater were evaluated. Emersion time has been previously described to influence the antioxidant response in juvenile *P. canaliculus*, increasing enzymatic antioxidant activity after 1–5 h of emersion at ~60% RH [19]. The efficacy of different hardening methods was evaluated by investigating the oxidative stress response, resettlement behaviour, respiration rate and survival of the juvenile mussels after transport and during immersion in seawater (i.e., recovery).

## 2. Materials and Methods

Juvenile Greenshell^TM^ mussels with a 1.2 ± 0.3 mm (±SD, *n* = 316) shell length were collected from the commercial hatchery SPATnz and transported to an adjacent research laboratory of the Cawthron Institute within Cawthron’s Aquaculture Park (Nelson, New Zealand). In the laboratory, the juveniles were separated, weighed and allocated to 112 circular sieves (8 cm diameter, 200 µm mesh size). The sieves were then randomly assigned to four experimental groups (26 sieves per hardening treatment and a control group not exposed to hardening). The experimental sieves were placed in a shallow tank with flowing 5 µm filtered seawater at 18 °C containing a standard 50:50 mix of microalgae (*Chaetoceros muelleri* and *Tisochrysis lutea*) at a concentration of 3–6 µg chlorophyll-*a* L^−1^. All sieves were supplied with microalgae during the first 48 h after collection before experimentation. The complete experiment was performed in three sequential phases: (1) a hardening phase, (2) a transport phase and (3) a recovery phase. Each experimental phase is explained in the section below.

### 2.1. Hardening, Transport and Recovery Phases

The hardening phase consisted of three levels of sub-lethal emersion conditions using airtight containers to hold the experimental sieves: (i) short emersion (1 h of emersion, followed by 1 h of immersion in seawater), (ii) long emersion (5 h of emersion, followed by 1 h of immersion) and (iii) intermittent emersion (3 cycles of 1 h of emersion followed by 1 h of immersion); there was also a control group in which mussel juveniles were not emersed. Hardening was performed in circular 750 mL airtight plastic containers containing desiccant silica gel to achieve 30–35% relative humidity (RH; mean ± SD = 33 ± 12%, *n* = 32). Relative humidity (RH) was monitored using RH loggers (Hygrochron, iButtonLink, Whitewater, WI, USA) which were set up to measure every 10 min during the emersion period. An RH of <60% has been shown to stimulate the activity of the main enzymatic antioxidants in juvenile *Perna canaliculus* exposed to emersion [19]. Experimental sieves containing mussels were patted dry with paper towels and randomly placed in containers allocated to each experimental treatment, with four replicate containers per treatment. The containers were tightly closed after the experimental sieves were placed inside and maintained in a temperature-controlled room at 18 °C for the duration of each hardening treatment (i.e., short, long or intermittent emersion). After each treatment, the experimental sieves were placed in flowing seawater for 1 h prior to the initiation of the transport phase of the experiment. Control sieves were maintained in flowing seawater while the experimental sieves were exposed to the hardening treatments.

The transport phase was simulated for both control and experimental sieves. Transport was carried out in similar circular, airtight containers as in the hardening phase but with a seawater-saturated cotton cloth on the inside of each container to achieve a high-relative-humidity environment during simulated transport (mean ± SD = 97 ± 3% RH, *n* = 720). The simulated transport lasted 20 h, and the containers were maintained at 18 °C.

The recovery phase was carried out after the transport phase. In this phase, the control and experimental sieves were immersed in flowing 18 °C seawater containing a mix of microalgae as described above for 24 h. During the first hour of recovery, resettlement behaviour (the adhesion of loose juveniles to coconut coir) and respiration rate (µmol O_2_ ash-free dry weight^−1^ h^−1^) were assessed, while the viability of the mussels was assessed after 24 h of the mussels being in recovery. It is important to note that the mussel juveniles that were used for the resettlement test and respiration rate assessment could be alive at the time of testing but lose viability within the following 24 h, and it was at that point that viability was determined.

### 2.2. Resettlement Behaviour Test

The resettlement behaviour of the juvenile mussels was tested immediately after simulated transport, at the start of the recovery phase (4 replicate sieves per treatment = 16 experimental sieves total). Resettlement was evaluated as previously described by South et al. [18]. Briefly, a pre-soaked coconut coir (20 cm long) was suspended inside a specialised conical tank commonly used for larval rearing [31]. These tanks were filled with 2 L of 5 µm filtered seawater (FSW) and bubbled from the bottom via a glass rod. After the simulated transport phase, juvenile mussels from the experimental sieves were removed using streams of freshwater and gentle finger pressure and immediately added to the respective tanks for resettlement. The juvenile mussels were allowed to resettle for 5 min, after which the coir was removed, bagged and frozen at −20 °C to facilitate mussel removal from the coir. The coir was processed by washing it with freshwater over a 500 µm sieve. Loose mussels were collected by washing the tanks with freshwater over a sieve. Mussels that settled onto the tanks and were resistant to washing with freshwater were removed and collected by freezing the tanks prior to washing them out over a 500 µm sieve. Mussels collected from the coir and tanks (loose and attached) were counted under a stereomicroscope (Olympus SZ61, Olympus, Melbourne, Australia), and resettlement was then calculated as the percentage of mussels attached in relation to the total number of mussels counted per tank [18].

### 2.3. Mussel Viability

The viability of the juvenile mussels was estimated at the end of the recovery phase, after 24 h in seawater (4 replicate sieves per treatment = 16 experimental sieves in total). After 24 h of recovery, the mussels were stained using the fast green method as an indication of fitness [32], with minor modifications as previously described by Delorme et al. [19]. In brief, staining was performed by placing mussels from each sieve into a 35 mL plastic container and adding freshwater to induce the closure of valves in healthy mussels. Then, one drop of concentrated fast green dye (0.1% *w*/*v*) was added, and the mussels were left immersed in the stain for 1 h. The mussels were then rinsed several times and frozen at −20 °C for a later assessment of unstained (healthy/live mussels) and stained (moribund/unhealthy or dead) mussels under a stereomicroscope.

### 2.4. Respiration Rate

The respiration rates of the juvenile mussels were determined at the start of the recovery phase to identify the metabolic response of the mussels during re-immersion after 20 h of transport (1 experimental sieve per treatment = 4 sieves total).

Between 5 and 10 juvenile mussels from each treatment were placed in each of 12 600–700 μL biochemical oxygen-demand glass vials (μ-BOD vials), with 316 mussels in 48 vials in total. The vials were filled with 0.3 µm FSW, sealed and incubated horizontally for 1.5–2 h at 18 °C. Seven μ-BOD vials were filled with FSW only as blanks to measure background oxygen concentration. After incubation, the oxygen concentration (µmol O_2_ L^−1^) in each μ-BOD vial was measured using a fibre-optic oxygen meter with an optical temperature sensor (FireSting^®^-02; PyroScience, Aachen, Germany), and data were acquired using the software Pyro Oxygen Logger V3.213 (PyroScience).

After measurements of oxygen consumption in each µ-BOD vial were obtained, the juvenile mussels were counted and weighed, and their ash-free dry weight (AFDW) was determined by drying the mussels in an oven for 24 h at 100 °C and then placing them in a muffle furnace at 450 °C for 6 h [23]. Respiration rates were then calculated per mg of the AFDW of the mussels in each vial and expressed as µmol of O_2_ per the AFDW of mussels per hour.

### 2.5. Biomarker Analyses

#### 2.5.1. Sampling for Biomarker Analyses

Experimental sieves assigned for biomarker analyses were sampled at the end of the hardening phase, immediately after the transport phase and during the recovery phases (1, 5 and 24 h after immersion in seawater). There were four replicate sieves per treatment per sampling point (80 experimental sieves in total). Sampling consisted of collecting sub-samples of ~130–140 mg of mussels (fresh wet weight, including shells) from each replicate sieve, placing the mussels in a 1.7 mL cryo-vial, flash-freezing the samples in liquid nitrogen and storing them at −80 °C until analyses. Samples were collected to determine oxidative damage (protein carbonyls (PCs), lipid hydroperoxides (LPs) and 8-hydroxydeoxyguanosine (8-OHdG)) and enzymatic antioxidant biomarkers (superoxide dismutase (SOD), catalase (CAT), glutathione peroxidase (GPx) and glutathione reductase (GR)).

#### 2.5.2. Macromolecule Extraction

Extractions of protein, lipids and DNA for the determination of oxidative damage and antioxidants in the samples were performed following a methodology described previously for juvenile *Perna canaliculus* [19]. Total protein was extracted on ice from the flash-frozen samples by adding 900 µL of ice-cold enzyme extraction buffer (100 mM potassium phosphate [pH 7.5] containing 50 mM NaCl, 0.1 mM Na_2_EDTA, 1% polyvinylpyrrolidone-40, 2 mM phenylmethylsulfonyl fluoride and 0.02% TritonX-100) to each sample and cold-homogenising the mixture for 2 cycles of 30 s at 1500 rpm (1600 MiniG^®^, SPEX^®^, Metuchen, NJ, USA) using zirconia/silica beads. The samples were then centrifuged for 15 min at 17,000× *g* and at 4 °C, and the supernatant (i.e., protein extract) was semi-purified using ultrafiltration and purification columns (Amicon^TM^, Merck, Rahway, NJ USA). The semi-purified protein extract was then washed by repeating the ultrafiltration step following the addition of a 50 mM potassium phosphate buffer (pH 7.2) and then reconstituted with 250 µL of a 50 mM potassium phosphate buffer (pH 7.2). The protein extract was then aliquoted into 2 × 1.7 mL microcentrifuge tubes, blown with oxygen-free nitrogen and stored at −80 °C until analysis. The two aliquots of protein extract were used to determine the level of protein carbonyls in the sample and to carry out antioxidant enzyme assays as described in Section 2.5.3 and Section 2.5.4, respectively.

Total lipids were extracted by adding 600 µL of methanol/chloroform (2:1 *v*/*v*) to each flash-frozen sample and cold-homogenised for 2 cycles of 30 s at 1500 rpm using zirconia/silica beads. The homogenised sample was left to stand for 5 min, and an extra 400 µL of chloroform was added and vortexed for 30 s. Then, 400 µL of MilliQ water was added, and the sample was vortexed again for 30 s. The samples were then centrifuged at ambient temperature for 30 s at 17,000× *g* (Prism^TM^R, Labnet, Edison, NJ, USA). Finally, the chloroform phase (bottom layer) was collected and transferred to a new 1.7 mL microcentrifuge tube, blown with oxygen-free nitrogen and stored at −80 °C until analysis.

DNA extraction was performed by using an ISOLATE II Genomic DNA Kit (Bioline, Memphis, TN, USA), with one minor modification. After the addition of the pre-lysis buffer, the flash-frozen samples were crushed and homogenised using a plastic tube pestle. The final DNA extracts were placed in a 1.7 mL microcentrifuge tube, blown with oxygen-free nitrogen and stored at −80 °C until analyses.

#### 2.5.3. Oxidative Damage Biomarkers

Protein content was determined using the Lowry protein assay [33]. Samples were diluted with potassium phosphate as required before analysis. The level of protein carbonyls (PCs) was determined via a reaction with 2.4-dinitrophenylhydrazine (DNPH), as described previously by Reznick and Packer [34], and expressed as nmols of carbonyls per mg of protein.

The level of lipid hydroperoxides (LPs) in the samples was determined by absorbance at 500 nm using the ferric thiocyanate method described previously by Mihaljevic et al. [35], which was adapted for measurement using a microtitre plate reader. A calibration curve with t-butyl hydroperoxide was used, and the LP content was calculated as nmol of lipid hydroperoxide per mg of fresh (wet) mussel weight (including shells).

PC and LP assays were carried out using a Victor 1420 Multilabel plate reader (Perkin Elmer Wallac, Buckinghamshire, UK) fitted with a temperature control cell (set to 25 °C) and an auto-dispenser. Data were acquired and processed using the WorkOut 2.0 software package (Perkin Elmer).

The level of oxidised DNA was calculated by quantifying the amount of 8-hydroxydeoxyguanosine (8-OHdG) present using high-performance liquid chromatography (HPLC), followed by the UV detection of guanine and the electrochemical detection (coulometric) of 8-OHdG as described previously for juvenile *P. canaliculus* [23].

#### 2.5.4. Enzymatic Antioxidant Biomarkers

The protein extract obtained from the total protein extraction described above was used to perform antioxidant enzyme assays of superoxide dismutase activity (SOD), catalase (CAT), glutathione peroxidase (GPx) and glutathione reductase (GR). The enzyme assays were performed as described previously for juvenile *P. canaliculus* [23]. SOD was determined using a Cayman Chemicals Superoxide Dismutase Assay Kit (Catalogue #706002, Ann Arbor, MI, USA) and the activity was expressed as units of SOD per mg of protein. CAT was assayed using a chemiluminescent method [36], as adapted previously by Janssens et al. [37] for 96-well microplates, and the activity was expressed as µmol per min per mg of protein. GPx activity was measured according to a spectrophotometric method [38] and expressed as nmol per min per mg of protein. GR was assayed using a microplate method [39] with minor modifications [23], and activity was expressed as nmol per min per mg of protein.

All enzymatic assays were carried out using a Perkin Elmer Wallac Victor 1420 multilabel counter as detailed above.

### 2.6. Statistical Analyses

All data were checked for normality and homoscedasticity using the Shapiro–Wilk and Brown–Forsythe tests, respectively. Raw respiration rate data were analysed with a one-way ANOVA, using the different hardening treatments as levels. Resettlement and viability percentage data were transformed (arcsine square root) and analysed using one-way ANOVAs.

The oxidative stress biomarker data collected were analysed with two-way ANOVAs using hardening treatment (control, short, long and intermittent emersion) and phase (hardening, transport, and 1 h recovery) as factors. The LPs, 8-OHdG, CAT and GR data collected here violated the homoscedasticity assumption; however, the data were analysed using two-way ANOVAs using a more conservative approach by reducing the alpha value to 0.01 [40].

In all the analyses, significant differences among groups were identified using Tukey pair-wise comparison tests with α = 0.05 except for LPs, 8-OHdG, CAT and GR (α = 0.01). Analyses were conducted using the statistical software Sigma Plot 14.0 (SYSTAT Software, Inc., Palo Alto, CA, USA).

## 3. Results

### 3.1. Resettlement Behaviour during Recovery

**Figure 1:**
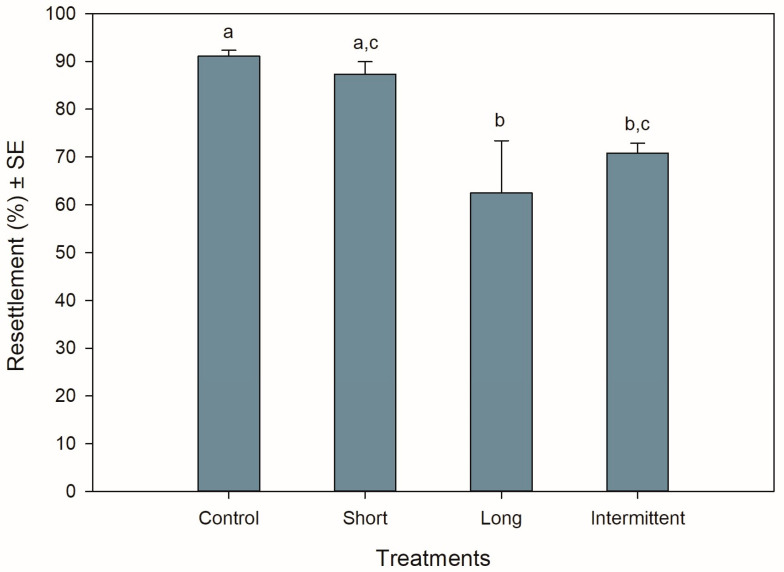
Resettlement behaviour of *Perna canaliculus* juveniles exposed to different hardening treatments after 20 h of simulated transport. Percentage of resettlement was measured during the recovery phase after transport, after 5 min of immersion in seawater. Control: no hardening treatment; short: 1 h of emersion followed by 1 h of immersion; long: 5 h of emersion followed by 1 h of immersion; intermittent: cyclic 1 h of emersion followed by 1 h of immersion (3 cycles total). Data represent mean resettlement ± standard error (SE, *n* = 4) values. Significant Tukey pair-wise comparisons (*p* < 0.05) are denoted by different lowercase letters above bars.

The resettlement behaviour of juvenile mussels starting the recovery phase was significantly different among treatments (ANOVA: F_3,12_ = 6.990, *p* = 0.006). Resettlement was >60% for all treatments, with mussels from the control group and short emersion treatment showing the highest resettlement percentages (Tukey: *p* = 0.917; Figure 1). Mussels from the control treatment had a resettlement 0.3- to 0.4-fold greater than mussels in the long (Tukey: *p* = 0.01) and intermittent (Tukey: *p* = 0.047) emersion treatments, respectively. Mussels exposed to the short emersion treatment also showed a 0.4-fold higher resettlement percentage than mussels exposed to the long emersion treatment (Tukey: *p* = 0.029) but were not significant when compared to juveniles exposed to the intermittent emersion treatment (Tukey: *p* = 0.137). Mussels exposed to the long and intermittent emersion treatments showed an 8% difference in resettlement percentage, with this difference not being statistically significant (Tukey: *p* = 0.801).

### 3.2. Mussel Viability at the End of Recovery

**Figure 2:**
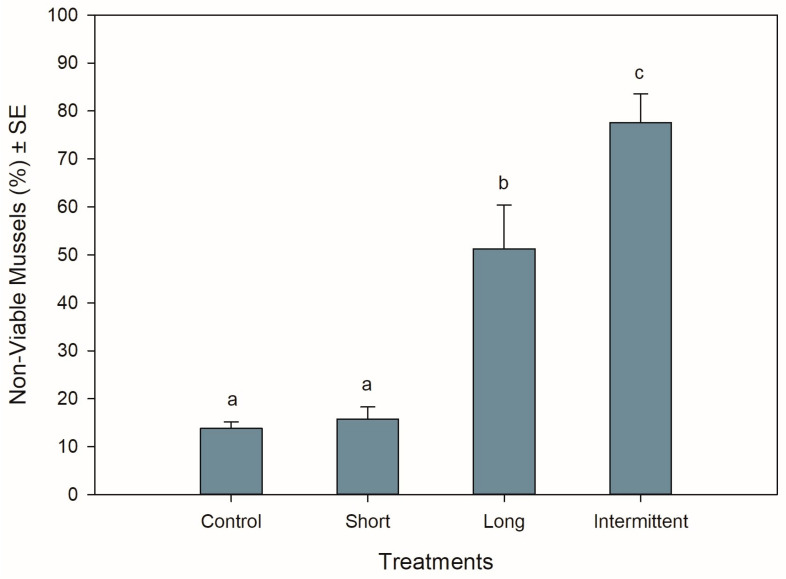
Viability of *Perna canaliculus* juveniles exposed to different hardening treatments prior to 20 h of simulated transport and followed by 24 h of recovery in seawater (*n* = 4). See the legend for Figure 1 for descriptions of treatments and data representation. Significant Tukey pair-wise comparisons (*p* < 0.05) are denoted by different lowercase letters above bars.

The different hardening treatments had significant effects on the viability of the mussels after 24 h of being in recovery following simulated transport (ANOVA: F_3,12_ = 29.517, *p* < 0.001). Mussels that were exposed to the short emersion treatment showed the lowest percentage of non-viable mussels (16%) together with the control mussels (14%) (Tukey: *p* = 0.992), suggesting that 84–86% of the mussels were in a good and viable condition after 24 h of recovery in seawater (Figure 2). In contrast, >50% of the mussels that were exposed to the long and intermittent emersion treatments were found to be non-viable (Figure 2), with the non-viability percentages from these treatments being 3- and 5-fold higher than mussels from both the control and short emersion treatments (Tukey: *p* < 0.004 for all comparisons). In addition, the level of viability observed for the long and intermittent emersion treatments was significantly different, with a difference of 27% in mussel viability between these treatments (Tukey: *p* = 0.03).

### 3.3. Respiration Rate during Recovery

**Figure 3:**
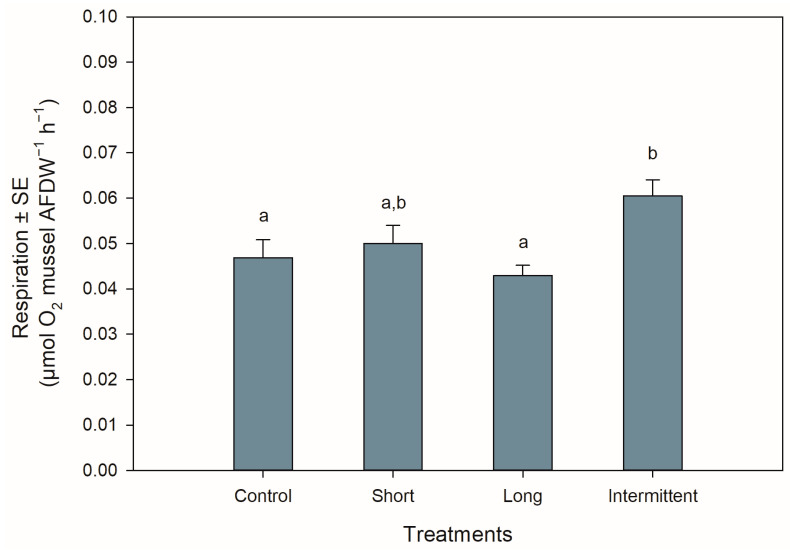
Respiration rates of *Perna canaliculus* juveniles exposed to different hardening treatments prior to 20 h of simulated transport. Respiration rate (µmol O_2_ mussel AFDW^−1^ h^−1^) was measured during the recovery phase after transport (*n* = 12). See the legend for Figure 1 for descriptions of treatments and data representation. Significant Tukey pair-wise comparisons (*p* < 0.05) are denoted by different lowercase letters above bars.

The respiration rates of the juvenile mussels at the start of the recovery phase were significantly different among treatments (ANOVA: F_3,44_ = 4.577, *p* = 0.007). Mussels exposed to the intermittent emersion treatment showed a ca. 25% increase in respiration rate compared to the control mussels (Tukey: *p* = 0.043) or mussels exposed to the long emersion treatment (Tukey: *p* = 0.005; Figure 3). Although the mussels from the intermittent emersion treatment showed a higher respiration rate than those mussels exposed to the short emersion treatment (Figure 3), the differences between means were not statistically significant (Tukey: *p* = 0.168). The respiration rates of the control mussels and those exposed to the short and long emersion treatments were not significantly different among them (Tukey: *p* > 0.491 for all comparisons).

### 3.4. Oxidative Damage and Enzymatic Antioxidant Biomarkers

**Figure 4:**
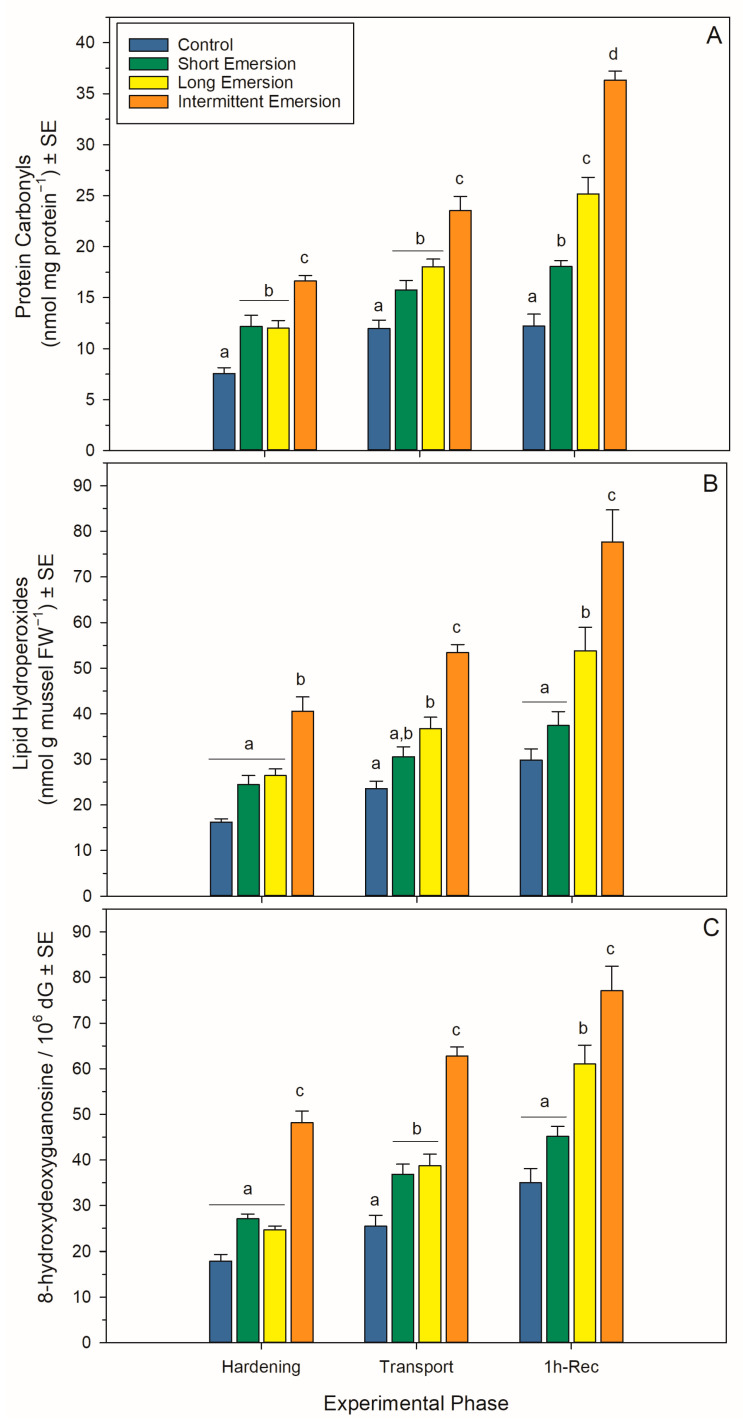
Oxidative damage biomarkers in *Perna canaliculus* juveniles. Mussels were exposed to different hardening treatments (hardening phase) prior to simulated transport for 20 h (transport phase, 0 h-Rec), followed by recovery (recovery phase) in seawater for 1 (1 h-Rec) (*n* = 4). See the legend for Figure 1 for descriptions of treatments and data representation. Significant Tukey pair-wise comparisons (*p* < 0.05) among treatment groups within each phase are denoted by different lowercase letters above bars.

**Figure 5:**
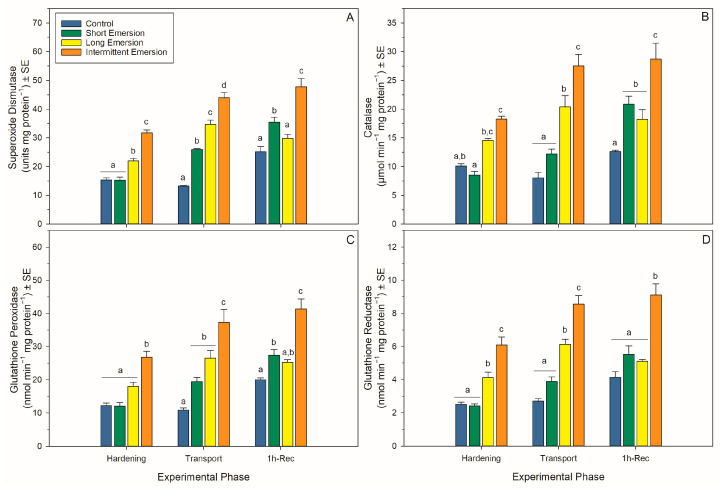
Enzymatic antioxidant biomarkers in *Perna canaliculus* juveniles. Mussels were exposed to different hardening treatments (Hardening Phase) prior to simulated transport for 20 h (Transport Phase, 0 h-Rec), followed by recovery (Recovery Phase) in seawater for 1 (1 h-Rec) (*n* = 4). See the legend for Figure 1 for descriptions of treatments and data representation. Significant Tukey pair-wise comparisons (*p* < 0.05) among treatment groups within each phase are denoted by different lowercase letters above bars.

There was a significant interaction effect of hardening treatment and experimental phase on levels of oxidative damage markers (PCs, LPs and 8-OHdG) and the activity of enzymatic antioxidants (SOD, CAT, GPx and GR) in juvenile *P. canaliculus* (Table S1, Figure 4 and Figure 5). Nonetheless, there was a strong effect of experimental phase on PC, LP and 8-OHdG levels for all hardening treatments (short, long and intermittent emersion) (Table S1, Figure 4). Below, detailed results are presented for the kinetics of the oxidative damage and enzymatic antioxidant biomarkers, separated by experimental phase (i.e., hardening, transport, 1 h of recovery, 5 h of recovery and 24 h of recovery) and biomarker type.

During the hardening phase, juvenile mussels exposed to the intermittent emersion treatment showed significantly higher levels of PCs, LPs and 8-OHdG compared to the control mussels (Tukey: *p* < 0.001 for all comparisons; Figure 4), while mussels exposed to the short and long emersion treatments had similar levels of PCs, LPs and 8-OHdG (Tukey: *p* > 0.928 for all comparisons; Figure 4). After hardening, the concentration of SOD and the levels of activity of CAT, GPx and GR were significantly higher in mussels exposed to the intermittent emersion treatment compared to the control mussels (Tukey: *p* < 0.001 for all comparisons; Figure 5). In contrast, the concentration of SOD and the levels of activity of CAT, GPx and GR in mussels exposed to the short emersion treatment were not significantly different from the control mussels (Tukey: *p* > 0.851 for all comparisons; Figure 5).

At the end of the transport phase (0 h of recovery in seawater), the mussels showed higher levels of PCs, LPs and 8-OHdG when exposed to the intermittent emersion treatment compared to mussels exposed to the control, short and long emersion treatments (Tukey: *p* < 0.004 for all comparisons; Figure 4). At this time, there was also a significant effect of experimental phase on SOD, CAT, GPx and GR activities at all hardening levels (Table S1, Figure 5). The concentrations of SOD in mussels that were exposed to all hardening treatments, including the control, were significantly different (Tukey: *p* < 0.001 for all comparisons), showing an increased concentration of SOD in the control mussels (lowest) compared to those exposed to the intermittent emersion treatment (highest) (Figure 5A). The levels of activity of GPx in mussels exposed to all hardening treatments were significantly higher than in control mussels (Tukey: *p* < 0.013 for all comparisons; Figure 5C). Similarly, CAT and GR activities at 0 h of recovery in seawater were also higher in mussels exposed to the long and intermittent emersion treatments (Tukey: *p* < 0.004 for all comparisons; Figure 5B,D). In addition, at 0 h of recovery, the activity levels of CAT and GR showed no significant differences between the control mussels and those exposed to the short emersion treatment (Tukey: *p* > 0.126; Figure 5B,D).

During re-immersion, after 1 h of the recovery phase, the levels of PCs, LPs and 8-OHdG were the highest in mussels exposed to the hardening treatments (short, long and intermittent emersion) (Figure 4), while the levels of PCs were significantly different among all hardening treatments and the control group (Tukey: *p* < 0.001; Figure 4A,C). In contrast, the levels of LPs and 8-OHdG after 1 h of recovery were not significantly different between mussels exposed to the control and short emersion treatments (Tukey: *p* > 0.059 for all comparisons; Figure 4B). In terms of enzyme activity in this recovery phase, the SOD concentration and GPx activity of mussels exposed to the short and intermittent emersion treatments were significantly higher than in control mussels (Tukey: *p* < 0.041 for all comparisons; Figure 5A,C). However, the SOD concentration and GPx activity of mussels exposed to the long emersion treatment were not different from the control mussels (Tukey: *p* > 0.124 for all comparisons; Figure 5A,C). The CAT activity for mussels for all treatments was significantly higher than that of the control mussels (Tukey: *p* < 0.031 for all comparisons; Figure 5B). However, mussels exposed to the short and long emersion treatments showed no statistical differences in CAT activity (Tukey: *p* > 0.541; Figure 5B); and the CAT activity for mussels exposed to the intermittent emersion treatment was significantly higher than the short and long emersion treatments and the control (Tukey: *p* < 0.001 for all comparisons; Figure 5B). The GR activity in mussels exposed to the intermittent emersion treatment was significantly higher than in the control mussels and those exposed to the short and long emersion treatments (Tukey: *p* < 0.001 for all comparisons; Figure 5D), with no significant differences in GR activity among the control, short and long emersion treatments (Tukey: *p* > 0.055 for all comparisons; Figure 5D).

## 4. Discussion

This study showed the overall performance of juveniles of the Greenshell^TM^ mussel, *Perna canaliculus*, exposed to different emersion times used as hardening treatments prior to simulated transport. The various hardening treatments resulted in varied physiological responses in the juvenile mussels, with the more severe treatments (long or intermittent emersion) resulting in low viability, low resettlement, greater oxidative damage and, in the case of some treatments, higher metabolism. In contrast, short emersion (1 h) did not cause significant stress to the mussels, which maintained respiration at control levels, showed an increased antioxidant response, showed greater resettlement behaviour than for more severe emersion treatments and remained viable after 24 h of immersion in seawater after transport. Hardening treatments can potentially be used as processes that can help mussels cope with reoxygenation stress during immersion, allowing them to recover. The stimulation of antioxidant defences using mild stress has been reported previously for several bivalve species [15,41,42], suggesting that exposing individuals to mild stressors may confer some degree of protection against long-lasting or more severe stress. Under stressful conditions, it is expected that the enzymatic antioxidant system in mussels is stimulated to cope with the stress, albeit with an associated energetic cost [43,44,45]. In this study, mussels exposed to the long and intermittent emersion treatments showed significant oxidative damage despite having elevated activities of antioxidant enzymes, and this most likely contributed to their high mortality. In addition, the allocation of resources to antioxidant protective mechanisms, as seen here in mussels exposed to the intermittent emersion treatment, could limit the availability of resources required for settlement and influence longer-term survival.

Reoxygenation stress after periods of anoxia or hypoxia is a common major stressor for intertidal marine species but also for aquaculture species that are emersed for transportation to their farm or market destinations. Intertidal bivalves are naturally adapted to cope with reoxygenation during high tide by stimulating antioxidant enzymes [46,47,48,49] and metabolic adjustments [50,51,52,53]. Bivalves also respond behaviourally (i.e., valve closure) when exposed to unfavourable conditions such as increasing temperatures or emersion. During valve closure, there is a shift from aerobic to anaerobic metabolism, resulting in a decrease in free radical production, protecting the individual from oxidative damage [54]. During emersion, mussels also use gaping behaviour to decrease their body temperature and facilitate gas exchange, helping mussels cope with long emersion periods [55,56,57]. In juvenile mussels, gaping behaviour has not been studied in detail, but previous research in *P. canaliculus* indicates that juvenile mussels gape during long emersion [19], potentially as a mechanism to sustain aerobic metabolism. In this study, the metabolism of juvenile *P. canaliculus* was not measured during emersion, but their respiration rates were measured upon immersion in seawater during recovery, showing that after 20 h of emersion and during reoxygenation, respiration rates in juvenile mussels exposed to the short emersion treatment were maintained at levels comparable to those observed in control mussels. This suggests that the short emersion treatment may be a good treatment to use as a hardening tool in juvenile *P. canaliculus* prior to transport. In other bivalve species, hypoxia has been shown to affect amino acid and fatty acid catabolism in the mussel *Mytilus edulis*, while reoxygenation has resulted in increased mitochondrial respiration but a decrease in the production of reactive oxygen species (ROS), indicating the ability of this mussel species to cope with reoxygenation stress [50]. In the mussel *M. galloprovincialis*, hypoxia can decrease the activity of antioxidant enzymes but increase the upregulation of antioxidant enzyme genes, returning both (enzymes and genes) to basal levels during reoxygenation, suggesting tolerance of this mussel to hypoxia and reoxygenation stress [58,59]. The clam *Scapharca inaequivalvis* has also shown increased levels of enzymatic antioxidants during reoxygenation following anoxic conditions [60]. In previous studies using *P. canaliculus* juveniles, air exposure resulted in increased oxidative damage, with the activity of antioxidant enzymes increasing rapidly during reoxygenation, showing that emersion time and relative humidity (RH) conditions during emersion are crucial for managing stress responses and performance in juvenile *P. canaliculus* [18,19].

It is important to note that in this study, hardening treatments were performed under reduced-RH conditions to challenge the mussels and therefore stimulate the production of protective mechanisms, including enzymatic antioxidants. In contrast, the transport phase of this experiment was performed under ideal conditions, with the RH being close to 100%, resulting in simulated transport that imparted no significant stress to the mussels. Therefore, control mussels that were not exposed to the hardening treatments showed reduced oxidative damage during the reoxygenation period compared to the mussels exposed to the hardening treatments. Although the control mussels in this study appeared to better manage oxidative damage during reoxygenation compared to mussels from the other treatments, the control mussels were exposed to unrealistic transport conditions with high humidity levels. Under “real-world” industry-relevant transport conditions, mussels may be less prepared to deal with reoxygenation stress, as well as other unfavourable conditions that mussels may encounter once they are deployed in farms at sea. Thus, it is possible that mussels that experience a mild pre-transport stress, like the 1 h emersion treatment seen in this study, are likely to be better prepared to cope with reoxygenation stress and other potential stressors after deployment.

Hardening methods have been explored in adult *P. canaliculus* to increase thermal tolerance [28,61] and to increase the shelf-life of adult mussels during live transport [56]. Physiological mechanisms that have been attributed to the successful hardening of bivalves to different conditions include improved mitochondrial respiration, increased antioxidant defence, changes in transcript levels and metabolic pathways and an increased synthesis of chaperone proteins [28,54,62]. Considering that more than 80% of the mussel industry in New Zealand relies on wild-caught juveniles that are collected in the far north of the country and then transported for over 72 h [5], it is relevant to determine if there are techniques that can potentially be used to help mussel juveniles cope with long emersion times, varying temperature conditions during emersion and reoxygenation stress. The current bottleneck for the New Zealand mussel industry is the massive loss of juvenile mussels within a few months of initial seeding [5,8,22]. The causes of these initial losses are complex, but increased stress exposure during transportation and immediately after seeding might affect the retention and survival of juvenile mussels. Therefore, transport hardening techniques, in conjunction with carefully managed transport conditions to reduce stress levels upon seeding, may play a critical role in successfully minimising losses of juvenile mussels.

Overall, this study showed that emersion hardening needs to be performed and managed carefully so as to stimulate protective mechanisms while avoiding overstressing the mussels, which could result in an excessively high energy demand. Exposing mussels to a mild stress may stimulate antioxidant defence mechanisms in preparation for a longer and stronger stress period that can occur when the mussels are transported. If transport practices are known to result in stressed juvenile mussels, appropriate emersion hardening treatments could help individuals cope with reoxygenation stress after they are deployed in the sea, having the potential to improve survival rates. However, this study clearly demonstrates that great care should be taken when choosing and applying hardening treatments. This study also shows that biomarkers associated with oxidative stress/antioxidant metabolism could be very valuable to ensure that emersion hardening treatments do not overstress the mussels and further reduce survival and that these biomarkers could be used as useful tools to help improve the management of stress during the transport of wild-caught juvenile mussels. This could be very beneficial to the shellfish industry by helping reduce the costs associated with mussel losses and by increasing harvest production.

## Data Availability

The data presented in this study are available upon reasonable request from the corresponding author.

## Supplementary Materials

The following supporting information can be downloaded at https://www.mdpi.com/article/10.3390/antiox13020198/s1, Table S1: Statistical analyses of oxidative damage and antioxidant biomarkers data for *Perna canaliculus* juveniles exposed to different hardening treatments (T) during the different experimental phases (P). Degrees of freedom (df), mean square (MS), F-ratio (F) and *p*-values are shown for each variable. Significant *p*-values (*p* < 0.05) are shown in bold.

## References

[1] Calderwood J., O’Connor N.E., Sigwart J.D., Roberts D. (2014). Determining optimal duration of seed translocation periods for benthic mussel (*Mytilus edulis*) cultivation using physiological and behavioural measures of stress. Aquaculture.

[2] Sievers M., Fitridge I., Bui S., Dempster T. (2017). To treat or not to treat: A quantitative review of the effect of biofouling and control methods in shellfish aquaculture to evaluate the necessity of removal. Biofouling.

[3] Novaes A.L.T., de Andrade G.J.P.O., dos Santos Alonço A., Magalhães A.R.M. (2019). Operational performance in aquaculture: A case study of the manual harvesting of cultivated mussels. Aquac. Eng..

[4] Kamermans P., Capelle J., Smaal A.C., Ferreira J.G., Grant J., Petersen J.K., Strand O. (2019). Provisioning of mussel seed and its efficient use in culture. Goods and Services of Marine Bivalves.

[5] South P.M., Delorme N.J., Skelton B.M., Floerl O., Jeffs A.G. (2022). The loss of seed mussels in longline aquaculture. Rev. Aquac..

[6] Capelle J.J., Wijsman J.W., Schellekens T., van Stralen M.R., Herman P.M., Smaal A.C. (2014). Spatial organisation and biomass development after relaying of mussel seed. J. Sea Res..

[7] Carton A., Jeffs A., Foote G., Palmer H., Bilton J. (2007). Evaluation of methods for assessing the retention of seed mussels (*Perna canaliculus*) prior to seeding for grow-out. Aquaculture.

[8] South P.M., Floerl O., Jeffs A.G. (2019). Magnitude and timing of seed losses in mussel (*Perna canaliculus*) aquaculture. Aquaculture.

[9] Hayden B.J., Woods C., Hatton S. (2007). Handling and storage conditions to optimise survival of mussel spat. N. Z. Mussel Farmers Assoc. Newsl..

[10] Hayden B.J., Woods C.M.C. (2011). Effect of water velocity on growth and retention of cultured Greenshell™ mussel spat, *Perna canaliculus* (Gmelin, 1791). Aquac. Int..

[11] Heasman K. (2013). Temperature and Humidity of Kaitaia Weed during Harvesting, Storage and Transport and Suggested Influences on Spat viability. Prepared for Marine Farmers Association.

[12] Jenewein B.T., Gosselin L.A. (2013). Ontogenetic shift in stress tolerance thresholds of *Mytilus trossulus*: Effects of desiccation and heat on juvenile mortality. Mar. Ecol. Prog. Ser..

[13] Hamilton H., Gosselin L. (2020). Ontogenetic shifts and interspecies variation in tolerance to desiccation and heat at the early benthic phase of six intertidal invertebrates. Mar. Ecol. Prog. Ser..

[14] Rivera-Ingraham G.A., Rocchetta I., Meyer S., Abele D. (2013). Oxygen radical formation in anoxic transgression and anoxia-reoxygenation: Foe or phantom? Experiments with a hypoxia tolerant bivalve. Mar. Environ. Res..

[15] Almeida E.A., Bainy A.C.D., Dafre A.L., Gomes O.F., Medeiros M.H., Di Mascio P. (2005). Oxidative stress in digestive gland and gill of the brown mussel (*Perna perna*) exposed to air and re-submersed. J. Exp. Mar. Biol. Ecol..

[16] Giannetto A., Maisano M., Cappello T., Oliva S., Parrino V., Natalotto A., De Marco G., Fasulo S. (2017). Effects of oxygen availability on oxidative stress biomarkers in the mediterranean mussel *Mytilus galloprovincialis*. Mar. Biotechnol..

[17] Falfushynska H., Piontkivska H., Sokolova I.M. (2020). Effects of intermittent hypoxia on cell survival and inflammatory responses in the intertidal marine bivalves *Mytilus edulis* and *Crassostrea gigas*. J. Exp. Biol..

[18] South P.M., Quirino M.N., LaDiega C., Delorme N.J. (2020). Emersion and relative humidity control resettlement success of juvenile marine mussels. Aquaculture.

[19] Delorme N.J., Burritt D.J., Ragg N.L.C., South P.M. (2021). Emersion and Relative humidity modulate stress response and recovery dynamics in juvenile mussels (*Perna canaliculus*). Metabolites.

[20] Jeffs A.G., Holland R.C., Hooker S.H., Hayden B.J. (1999). Overview and bibliography of research on the greenshell mussel, *Perna canaliculus*, from New Zealand waters. J. Shellfish. Res..

[21] Skelton B.M., Jeffs A.G. (2021). The loss of spat following seeding onto coastal Greenshell™ mussel (*Perna canaliculus*) farms. Aquaculture.

[22] Skelton B.M., South P.M., Jeffs A.G. (2022). Inefficiency of conversion of seed into market-ready mussels in New Zealand’s Greenshell™ mussel (*Perna canaliculus*) industry. Aquaculture.

[23] Delorme N., Biessy L., South P., Zamora L., Ragg N., Burritt D. (2020). Stress-on-stress responses of a marine mussel, *Perna canaliculus*: Food limitation reduces the ability to cope with heat stress in juveniles. Mar. Ecol. Prog. Ser..

[24] Supono S., Dunphy B., Jeffs A. (2020). Retention of green-lipped mussel spat: The roles of body size and nutritional condition. Aquaculture.

[25] Supono S., Yu X., Skelton B.M., McKay W.J., Jeffs A. (2022). Effect of starvation on the nutritional condition of juvenile green-lipped mussels of different sizes. Aquaculture.

[26] Bowler K. (2005). Acclimation, heat shock and hardening. J. Therm. Biol..

[27] Clegg J., Uhlinger K., Jackson S., Cherr G., Rifkin E., Friedman C. (1998). Induced thermotolerance and the heat shock protein-70 family in the Pacific oyster *Crassostrea gigas*. Mol. Mar. Biol. Biotechnol..

[28] Dunphy B., Ruggiero K., Zamora L., Ragg N. (2018). Metabolomic analysis of heat-hardening in adult green-lipped mussel (*Perna canaliculus*): A key role for succinic acid and the GABAergic synapse pathway. J. Therm. Biol..

[29] Demers A., Guderley H. (1994). Acclimatization to intertidal conditions modifies the physiological response to prolonged air exposure in *Mytilus edulis*. Mar. Biol..

[30] Anestis A., Pörtner H.O., Michaelidis B. (2010). Anaerobic metabolic patterns related to stress responses in hypoxia exposed mussels *Mytilus galloprovincialis*. J. Exp. Mar. Biol. Ecol..

[31] Ragg N.L., King N., Watts E., Morrish J. (2010). Optimising the delivery of the key dietary diatom *Chaetoceros calcitrans* to intensively cultured Greenshell™ mussel larvae, *Perna canaliculus*. Aquaculture.

[32] Webb S., Heasman K. (2006). Evaluation of fast green uptake as a simple fitness test for spat of *Perna canaliculus* (Gmelin, 1791). Aquaculture.

[33] Fryer H.J., Davis G.E., Manthorpe M., Varon S. (1986). Lowry protein assay using an automatic microtiter plate spectrophotometer. Anal. Biochem..

[34] Reznick A.Z., Packer L. (1994). Oxidative damage to proteins: Spectrophotometric method for carbonyl assay. Methods Enzymol..

[35] Mihaljević B., Katušin-Ražem B., Ražem D. (1996). The reevaluation of the ferric thiocyanate assay for lipid hydroperoxides with special considerations of the mechanistic aspects of the response. Free. Radic. Biol. Med..

[36] Maral J., Puget K., Michelson A. (1977). Comparative study of superoxide dismutase, catalase and glutathione peroxidase levels in erythrocytes of different animals. Biochem. Biophys. Res. Commun..

[37] Janssens B.J., Childress J.J., Baguet F., Rees J.-F. (2000). Reduced enzymatic antioxidative defense in deep-sea fish. J. Exp. Biol..

[38] Paglia D.E., Valentine W.N. (1967). Studies on the quantitative and qualitative characterization of erythrocyte glutathione peroxidase. J. Lab. Clin. Med..

[39] Cribb A.E., Leeder J., Spielberg S.P. (1989). Use of a microplate reader in an assay of glutathione reductase using 5,5′-dithiobis(2-nitrobenzoic acid). Anal. Biochem..

[40] Underwood A.J. (1996). Experiments in Ecology: Their Logical Design and Interpretation Using Analysis of Variance.

[41] Freire C.A., Welker A.F., Storey J.M., Storey K.B., Hermes-Lima M., Abele D., Vázquez-Medina J.P., Zenteno-Savín T. (2012). Oxidative stress in estuarine and intertidal environments (temperate and tropical). Oxidative Stress in Aquatic Ecosystems.

[42] David E., Tanguy A., Pichavant K., Moraga D. (2005). Response of the Pacific oyster Crassostrea gigas to hypoxia exposure under experimental conditions. FEBS J..

[43] Delorme N.J., Venter L., Rolton A., Ericson J.A. (2021). Integrating animal health and stress assessment tools using the green-lipped mussel *Perna canaliculus* as a case study. J. Shellfish. Res..

[44] Sokolova I.M., Sukhotin A.A., Lannig G., Abele D., Vázquez-Medina J.P., Zenteno-Savín T. (2012). Stress effects on metabolims and energy budgets in mollusks. Oxidative Stress in Aquatic Ecosystems.

[45] Istomina A., Yelovskaya O., Chelomin V., Karpenko A., Zvyagintsev A. (2021). Antioxidant activity of Far Eastern bivalves in their natural habitat. Mar. Environ. Res..

[46] de Almeida E.A., Bainy A.C.D. (2006). Effects of aerial exposure on antioxidant defenses in the brown mussel *Perna perna*. Braz. Arch. Biol. Technol..

[47] Istomina A., Belcheva N., Chelomin V. (2013). Antioxidant system of the intertidal mollusk *Littorina kurila* in its natural habitat. J. Environ. Sci. Eng. A.

[48] Welker A.F., Moreira D.C., Campos É.G., Hermes-Lima M. (2013). Role of redox metabolism for adaptation of aquatic animals to drastic changes in oxygen availability. Comp. Biochem. Physiol. Part A Mol. Integr. Physiol..

[49] Giraud-Billoud M., Rivera-Ingraham G.A., Moreira D.C., Burmester T., Castro-Vazquez A., Carvajalino-Fernández J.M., Dafre A., Niu C., Tremblay N., Paital B. (2019). Twenty years of the ‘Preparation for Oxidative Stress’(POS) theory: Ecophysiological advantages and molecular strategies. Comp. Biochem. Physiol. Part A Mol. Integr. Physiol..

[50] Sokolov E.P., Adzigbli L., Markert S., Bundgaard A., Fago A., Becher D., Hirschfeld C., Sokolova I.M. (2021). Intrinsic mechanisms underlying hypoxia-tolerant mitochondrial phenotype during hypoxia-reoxygenation stress in a marine facultative anaerobe, the blue mussel *Mytilus edulis*. Front. Mar. Sci..

[51] Sokolov E.P., Markert S., Hinzke T., Hirschfeld C., Becher D., Ponsuksili S., Sokolova I.M. (2019). Effects of hypoxia-reoxygenation stress on mitochondrial proteome and bioenergetics of the hypoxia-tolerant marine bivalve *Crassostrea gigas*. J. Proteom..

[52] Adzigbli L., Sokolov E.P., Ponsuksili S., Sokolova I.M. (2022). Tissue- and substrate-dependent mitochondrial responses to acute hypoxia–reoxygenation stress in a marine bivalve (*Crassostrea gigas*). J. Exp. Biol..

[53] Haider F., Falfushynska H.I., Timm S., Sokolova I.M. (2020). Effects of hypoxia and reoxygenation on intermediary metabolite homeostasis of marine bivalves Mytilus edulis and Crassostrea gigas. Comp. Biochem. Physiol. Part A Mol. Integr. Physiol..

[54] Georgoulis I., Feidantsis K., Giantsis I.A., Kakale A., Bock C., Pörtner H.O., Sokolova I.M., Michaelidis B. (2021). Heat hardening enhances mitochondrial potential for respiration and oxidative defence capacity in the mantle of thermally stressed *Mytilus galloprovincialis*. Sci. Rep..

[55] Nicastro K.R., Zardi G.I., McQuaid C.D., Pearson G.A., Serrão E.A. (2012). Love Thy Neighbour: Group Properties of gaping behaviour in mussel aggregations. PLoS ONE.

[56] Zamora L.N., Ragg N.L., Hilton Z., Webb S.C., King N., Adams S. (2019). Emersion survival manipulation in Greenshell™ mussels (*Perna canaliculus*): Implications for the extension of live mussels’ shelf-life. Aquaculture.

[57] Powell J., Ragg N., Dunphy B. (2017). Phenotypic biomarkers in selectively-bred families of the Greenshell™ mussel (*Perna canaliculus*): Anaerobic enzyme and shell gape behaviour as biomarkers of prolonged emersion tolerance. Aquaculture.

[58] Andreyeva A., Gostyukhina O.L., Kladchenko E.S., Afonnikov D.A., Rasskazov D.A., Lantushenko A.O., Vodiasova E.A. (2021). Hypoxia exerts oxidative stress and changes in expression of antioxidant enzyme genes in gills of *Mytilus galloprovincialis* (Lamarck, 1819). Mar. Biol. Res..

[59] Gostyukhina O., Yu A.A., Chelebieva E., Vodiasova E., Lantushenko A., Kladchenko E. (2022). Adaptive potential of the Mediterranean mussel *Mytilus galloprovincialis* to short-term environmental hypoxia. Fish Shellfish. Immunol..

[60] Foschi J., Mancini G., Fabbri M., Rosmini R., Serrazanetti G., Monari M. (2011). Antioxidant defences role during post anoxic recovery in bivalve mollusc *Scapharca inaequivalvis*. J. Biol. Res.-Boll. Della Soc. Ital. Di Biol. Sper..

[61] Ravishankar S., Ragg N., Delorme N., Dunphy B. (2023). Thermotolerance of Greenshell™ mussel spat (*Perna canaliculus*) improved by prickly pear (*Opuntia ficus indica*) treatments. Aquaculture.

[62] Zhang W., Dong Y. (2021). Membrane lipid metabolism, heat shock response and energy costs mediate the interaction between acclimatization and heat-hardening response in the razor clam *Sinonovacula constricta*. J. Exp. Biol..

