# Supplementary material for: Oxidative Damage and Antioxidants as Markers for the Selection of Emersion Hardening Treatments in GreenshellTM Mussel Juveniles (Perna canaliculus)
Source: Antioxidants (Basel). 2024 Feb 4;13(2):198. doi: 10.3390/antiox13020198 (PMC10886077; doi:10.3390/antiox13020198)
Supplement: Supplementary file 1 [file antioxidants-13-00198-s001.zip › antioxidants-2819855-supplementary.pdf]

Table S1. Statistical analyses of oxidative damage and antioxidant biomarkers data for *Perna canaliculus* juveniles exposed to different hardening treatments (T) during the different experimental phases (P). Degrees of freedom (df), mean square (MS), F-ratio (F) and p-values are shown for each variable. Significant p-values (p < 0.05) are shown in **bold**.

**Protein Carbonyls**	**df**	**MS**	**F**	**p-value**
Treatment (T)	3	469.67	121.66	**<0.001**
Phase (P)	2	471.36	122.10	**<0.001**
T × P	6	54.98	14.24	**<0.001**
Residual	36	3.86		
**Lipid Hydroperoxides**	**df**	**MS**	**F**	**p-value**
Treatment (T)	3	2557.45	61.91	**<0.001**
Phase (P)	2	2101.46	50.87	**<0.001**
T × P	6	146.93	3.56	**0.007**
Residual	36	41.31		
**8-Hydroxydeoxyguanosine**	**df**	**MS**	**F**	**p-value**
Treatment (T)	3	2845.87	93.58	**<0.001**
Phase (P)	2	2528.89	83.16	**<0.001**
T × P	6	90.89	2.99	**0.018**
Residual	36	30.41		
**Superoxide Dismutase**	**df**	**MS**	**F**	**p-value**
Treatment (T)	3	1123.85	132.11	**<0.001**
Phase (P)	2	736.88	86.62	**<0.001**
T × P	6	91.39	10.74	**<0.001**
Residual	36	8.51		
**Catalase**	**df**	**MS**	**F**	**p-value**
Treatment (T)	3	467.11	62.19	**<0.001**
Phase (P)	2	210.82	28.07	**<0.001**
T × P	6	45.21	6.02	**<0.001**
Residual	36	7.51		
**Glutathione Peroxidase**	**df**	**MS**	**F**	**p-value**
Treatment (T)	3	937.09	66.86	**<0.001**
Phase (P)	2	506.13	36.11	**<0.001**
T × P	6	45.17	3.22	**0.012**
Residual	36	14.02		
**Glutathione Reductase**	**df**	**MS**	**F**	**p-value**
Treatment (T)	3	52.72	96.21	**<0.001**
Phase (P)	2	20.13	36.74	**<0.001**
T × P	6	2.34	4.28	**0.002**
Residual	36	0.55		
